# Modelling complex sports adaptation with electrocardiography: analysis of standard ECG, heart rate variability and QRST integral changes in elite athletes

**DOI:** 10.1186/s13102-026-01901-8

**Published:** 2026-07-24

**Authors:** Dóra Boroncsok, Máté Babity, Márk Zámodics, Anna Menyhárt-Hetényi, Gergely Tuboly, György Kozmann, Regina Benkő, Alexandra Fábián, Zsuzsanna Ladányi, Dorottya Balla, Hajnalka Vágó, Attila Kovács, Béla Merkely, Orsolya Kiss

**Affiliations:** 1 Heart and Vascular Center, Semmelweis University, Városmajor street 68, Budapest, 1122 Hungary; 2 Department of Sports Medicine, Semmelweis University, Városmajor street 68, Budapest, 1122 Hungary; 3 State Heart Hospital, Gyógy square 2, Balatonfüred, 8230 Hungary; 4 Department of Electrical Engineering and Information Systems, University of Pannonia, Egyetem street 10, Veszprém, 8200 Hungary

**Keywords:** Athlete’s heart, Electrocardiography, Heart rate variability, Ventricular heterogeneity, Sports adaptation

## Abstract

**Background:**

Basic resting electrocardiographic changes caused by regular intense exercise are known characteristics of the athlete’s heart. Through detailed analysis of short-term ECG recordings, our aim was to study electrocardiographic, heart rate variability, and ventricular heterogeneity changes of the athlete’s heart as a function of factors influencing sports adaptation.

**Methods:**

Analysis of 1-minute resting lead I ECG recordings was used to determine standard ECG parameters, time-domain indicators of heart rate variability, and mean and standard deviation of QRST integrals. Statistical analysis was performed in Python development environment.

**Results:**

500 healthy athletes (age: 19[16–24]y, male: 55%, training: 16.7 ± 6.5 h/w) were compared with 100 non-athlete controls (age: 21[17–23]y, male: 51%). Athletes had lower heart rate (68[61–78]vs.82[73–91]BPM, *p* < 0.001), increased p-wave amplitude (89[71–108]vs.80[62–96]µV, *p* < 0.001), PQ interval (152[140–168]vs.142[132–152]ms, *p* < 0.001), QRS width (90[82–100]vs.82[78–90]ms, *p* < 0.001), T-wave amplitude (257[198–311]vs.195[146–248]µV, *p* < 0.001), SDNN (64.7[47.2–87.2]vs.50.6[36.5–66.3]ms, *p* < 0.001), RMSSD (52.6[34.8–78.3]vs.36.4[26.8–52.6]ms, *p* < 0.001), pNN50 (31.1[12.7–50.9]vs.14.7[4.9–31.6]%, *p* < 0.001) and mean QRST integral (34.9[28.3–43.4]vs.26.1[19.7–33.9]mV*ms, *p* < 0.001) compared to controls. Mixed athletes had wider QRS compared to power athletes (94[90–102]vs.88[84–96]ms, *p* < 0.005). Male athletes had lower QTc, increased PQ interval, QRS width, T-wave amplitude, mean QRST integral, and its deviation. In the control group, females had higher resting heart rate and QTc duration, while AVNN and the relative deviation of QRST integral were lower. Many of the above parameters also showed weak correlation with age and weekly exercise hours.

**Conclusions:**

Our findings indicate that a 1-minute lead I ECG recording can detect electrical differences between athletes and non-athlete controls and may provide a practical framework for future longitudinal studies of athletic cardiac adaptation.

**Graphical Abstract:**

**Figure:**
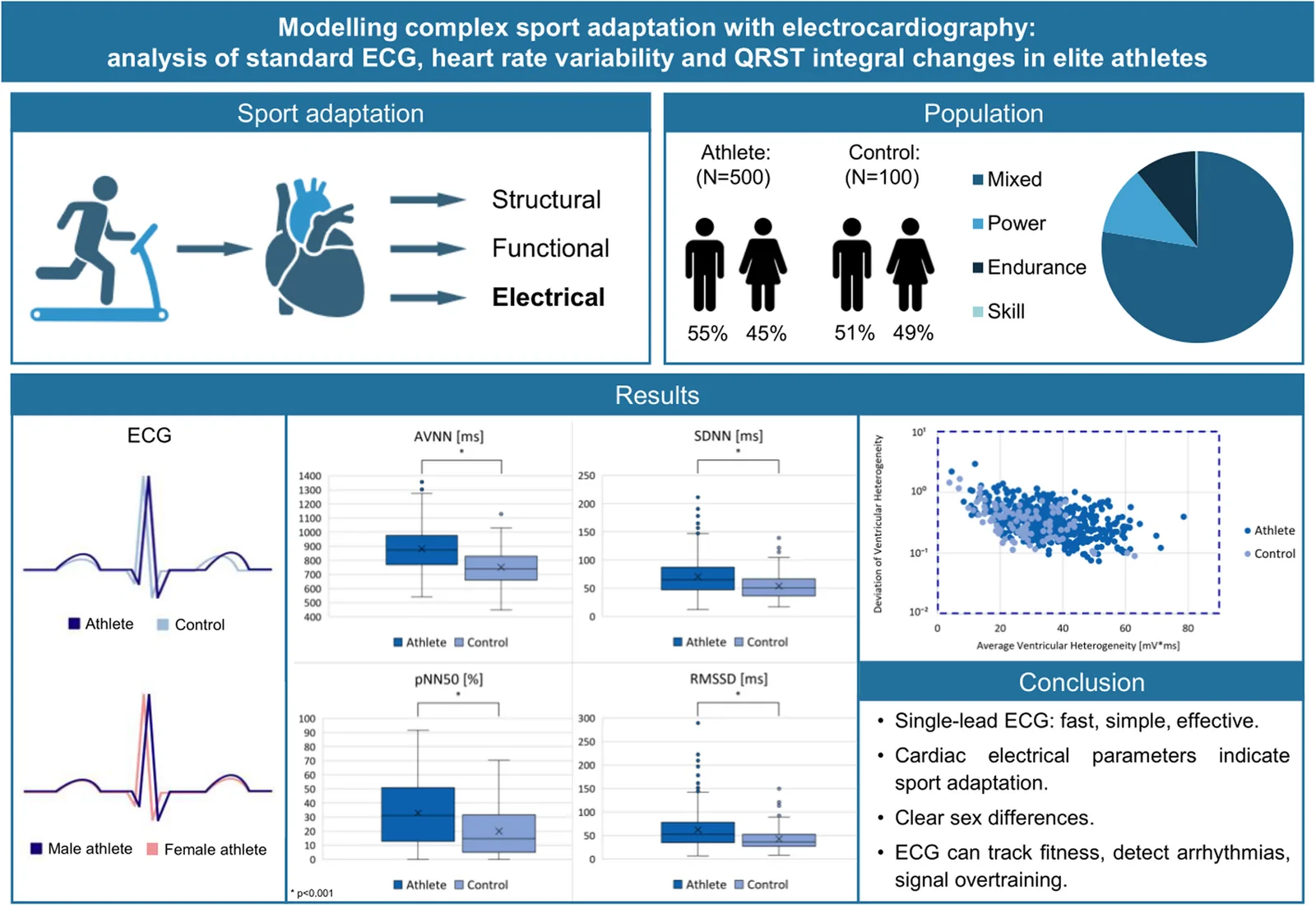
Abbreviations: AVNN: average N-N interval, SDNN: standard deviation of N-N intervals, RMSSD: root mean square of successive N-N differences, pNN50: proportion of N-N intervals that differ by at least 50 ms

## Background

As our organ systems adapt to regular intensive physical exercise, daily sports activities induce important changes in the human body. This process is even more significant in the case of elite athletes [1]. Besides the well-known effects on the musculoskeletal system, regular sports activity also produces significant changes in the cardiovascular system [2, 3]. In addition, it induces positive neurological and psychological changes [4]; and besides the central and peripheral nervous systems, it also positively modifies autonomic nervous system function [5, 6]. Favourable changes in hormonal status may also be observed [7], while these neurological and hormonal changes also significantly influence cardiovascular health and sports adaptation.

Due to the increased physical stress during regular exercise, the cardiovascular system needs to adapt to the extra workload, developing the athlete’s heart, which is the result of the complex interaction of the above-mentioned processes. Cardiac sports adaptation takes place at three strongly related levels: structural, functional, and electrical remodelling of the heart. The mechanisms of structural and functional remodelling are already well characterized by a large amount of literature [6, 8–12], in contrast to electrical remodelling, which is a less defined, although not less important, feature of the athlete’s heart.

In the athletes’ heart, major structural remodelling changes can be observed. The increase in myocardial muscle mass leads to eccentric or concentric left ventricular hypertrophy, depending on the type of sport. Long-term dynamic endurance sports like swimming, cycling, or rowing commonly cause eccentric hypertrophy, while static power sports like weightlifting or wrestling are reported to cause rather concentric hypertrophy [8]. The dilatation of the left ventricle with a significant increase of the end-diastolic volume is a common sports adaptation sign, especially in long-term dynamic endurance sports. Structural remodelling is not limited to the left ventricle, the two ventricles adapt together, with wall thickening and dilatation of the right ventricle [9, 13]. Moreover, as the increased volume load induced by exercise affects not only the ventricles but also the atria, long-term sports adaptation processes often result in the dilatation of both atria [10]. 

Besides structural remodelling, the electrical system also adapts to the increased load. This process can well be monitored by ECG recordings, which show changes due to myocardial muscle mass and chamber size increase and due to autonomic sports adaptation, as well as sport-related alterations down to the level of ionic channels. Sports adaptation causes certain electrocardiographic changes that may refer to pathological processes in those who do not exercise regularly, while they are considered physiological variants in athletes. Such changes are the increased QRS voltage or incomplete right bundle branch block signs due to ventricular hypertrophy, or sinus bradycardia, early repolarization, first degree atrioventricular block, and Mobitz I. second degree atrioventricular block due to the autonomic changes [14–17]. In healthy athletes, autonomic balance is shifted towards the parasympathetic direction at rest as a sign of autonomic sports adaptation. Electrocardiography can also be used to examine time and frequency domain indicators of heart rate variability (HRV), the phenomenon describing short and long-term changes in beat-to-beat R-R intervals. Heart rate variability depends on both sympathetic and parasympathetic tones and their ratio. Due to pathological states like overtraining, HRV parameters characterizing the sympathetic tone could show an elevation [18, 19]. 

Another less-known parameter that reflects the electrical state of the heart and can be assessed by analysing ECG recordings is ventricular heterogeneity. Parameters of ventricular heterogeneity characterise the phenomenon that myocardial cells do not depolarise and repolarise at the same time, but at different moments of time. Pathological changes in ventricular heterogeneity refer to increased differences in the repolarisation of certain myocardial cells or regions and can be a predictor of the development of malignant arrhythmias [20]. This process can be well studied by calculating the area under the QRST curve as the QRST integral, which depends on the differences in the area under the action potential curves and the electrical characteristics of distinct myocardial cells [21, 22]. No data are available in the literature regarding the changes in ventricular heterogeneity due to physiological sports adaptation processes of the heart.

Functional adaptation of the athletes’ heart contributes to optimising cardiovascular performance during sports activities. This complex process is strongly connected to the structural and electrical changes detailed above, while the autonomic nervous system adaptations, such as increased resting parasympathetic tone and increased sympathetic activity during exercise, also play an important role in it. Increased resting parasympathetic tone, in addition to reducing resting heart rate, also increases the relaxation ability of the heart, thereby improving the diastolic function. On the other hand, increased sympathetic tone during exercise has positive inotropic, chronotropic, and dromotropic effects. During intensive physical exercise, enhanced stroke volume is essential to improve cardiopulmonary performance and is mainly achieved through larger cavity sizes, improved contractility of the myocardium, and a reduction in end-systolic volume [6]. 

Several approaches have been proposed for assessing electrical sports adaptation, including standard 12‑lead ECG, long‑term Holter monitoring, and conventional 5‑minute or 24‑hour HRV analysis, each providing valuable but logistically demanding information on training‑related electrical remodelling. However, these methods are less suitable for frequent large‑scale or field‑based athlete monitoring because they require specialized equipment, longer recording times, and expert interpretation. In contrast, simple single‑lead, ultra‑short‑term ECG recordings obtained with portable devices may offer a practical alternative for repeated assessment of key electrical adaptation markers, but their utility in elite athletes has not yet been systematically characterized.

Our study was based on previous data showing that the electrical characteristics of the hearts of athletes and non-athletes significantly differ. The primary aim of the present study was to characterize electrical sports adaptation using standard ECG parameters, ultra-short-term HRV indices, and QRST-integral-derived markers of ventricular heterogeneity. As a secondary objective, we investigated whether these parameters could be obtained using a simple portable single-lead ECG platform that may facilitate future large-scale or longitudinal athlete examinations.

Following basic comparisons between athletes and controls, we also compared the measured and calculated parameters for female and male athletes, assuming that there are differences between the two sexes in cardiac electrical adaptation to exercise. Finally, we analysed exploratory the parameters by sport type to see whether, like structural changes, the adaptation of the electrical system also shows disparities regarding sport activity.

## Methods

### Participants

In a 7-year period from 2016, elite athletes and age and sex matched non-athlete controls were recruited to the current study. For inclusion in any of the groups, the participants needed to be asymptomatic and free from any significant health conditions (with particular regard to significant cardiovascular diseases, infections, metabolic diseases, and psychological issues) that could have influenced the results. Athletes were considered elite if they were national team athletes, Olympians, or professional athletes who regularly trained for more than 10 h per week. During data processing, competitive sport activities of the athletes were characterised as skill, power, mixed, and endurance sports regarding the sport classification of the European Society of Cardiology (ESC) [2]. Non-athlete controls were required to train less than 5 h per week and to have no previous official competitive athletic background. Individuals with pathological findings due to the cardiovascular evaluation were excluded from the study. To ensure similar conditions for all participants, the measurements took place during the daytime, at least 12 h after the last training or competition, in the same laboratory circumstances.

Prior to the study, all participants – in case of underage participants (< 18-year-old) also their parents or legal guardians – gave written informed consent to the examinations, and the Scientific and Research Committee of the Medical Research Council of Hungary approved the study (No.: IV/9697-1/2020/EKU) according to the Ethical Guidelines of the Helsinki Declaration and to Good Clinical Practice.

All participants included in the study completed an extended sports cardiology screening programme, which was further extended with our specific ECG analysis. The extended sports cardiology examination included detailed medical history recording, laboratory blood tests, resting blood pressure measurement, resting 12-lead ECG recording, bioimpedance body composition measurements, resting echocardiography, and maximal intensity cardiopulmonary exercise testing.

### ECG recording and analysis

For the specific analysis, ECG recordings were made using three identical small portable devices developed, validated, and medically licensed in Hungary called Wiwe (myWIWE Diagnosztika Kft, Hungary) [23–25]. This mobile equipment provides one-minute, single channel standard lead I ECG recordings in a resting sitting position. All ECG recordings included in the present analysis were obtained during a single study visit under standardized resting conditions. Repeated recordings were not included in the present analysis. Subsequent analysis was made from raw data extracted from the device by mathematical modeling [21]. After the detection and exclusion of all premature beats, an averaged majority ECG curve was calculated, and standard ECG parameters (heart rate, P-wave amplitude, PQ interval, QRS width, QRS amplitude, T-wave amplitude, and QTc duration by Bazett’s formula) were determined from the standard lead I recordings.

### Heart rate variability analysis

Subsequent analysis of time domain heart rate variability (HRV) parameters of the above-described one-minute standard lead I ECG recordings was carried out [26]. The examined HRV time domains were as follows: average N-N interval (AVNN), standard deviation of N-N intervals (SDNN), root mean square of successive N-N differences (RMSSD), proportion of N-N intervals that differ by at least 50 milliseconds from each other compared to the total NN intervals (pNN50). Participants were instructed to remain seated quietly and breath normally during ECG acquisition. However, the breathing pattern was not paced or standardized. The ECG signal was processed automatically once the measurement was complete. The first step in this process involved applying a 0.5 Hz high-pass filter and a 40 Hz low-pass filter to the signal in order to reduce noise. After noise filtering, the algorithm identified individual heartbeats (QRS detection) and then classified them. All premature beats were automatically detected and excluded prior to HRV analysis. Only recordings with adequate signal quality allowing reliable R-wave detection were included in the analysis.

### Ventricular heterogeneity analysis

The third group of analysed variables was the parameters of ventricular heterogeneity [27]. Based on the standard ECG lead I data recorded by the Wiwe device, 1-minute average QRST integral and relative deviation of the QRST integrals were determined, analysed, and plotted using the waveform thresholds obtained from the averaged cycle. QRST-integral calculations were performed using the previously validated proprietary algorithm integrated into the ECG device software [28]. Signal preprocessing, beat averaging, and integral calculations were performed automatically according to the methodology described in the original validation studies. Specifically: (1) after QRS detection and classification, all premature beats were automatically excluded from the analysis; (2) an averaged majority ECG curve was calculated from the normal sinus beats of the 1-minute recording; (3) waveform thresholds (onset and offset of the P, QRS, and T waves) were determined from this averaged cycle using the established device algorithm; (4) the QRST integral was calculated as the area under the QRST complex of the averaged cycle, expressed in mV*ms; (5) the relative deviation of QRST integral was calculated as the standard deviation of individual QRST integrals divided by the mean QRST integral. The algorithm was applied in a standardised, fully automated manner to all recordings without manual adjudication of individual waveform boundaries. The method is based on the established relationship between QRST integrals and myocardial repolarization heterogeneity and includes the analysis of the temporal variability of consecutive QRST integral maps [22]. According to the data of a previous study carried out on a large number of patients with known malignant ventricular arrhythmias, patients with severe heart failure predisposing to malignant arrhythmias, and healthy controls, the examined individuals were placed in one of the healthy, intermediate, and malignant arrhythmia-prone groups by the Wiwe device [22]. In the original validation study, the intermediate category consisted of patients with reduced left ventricular ejection fraction without documented malignant ventricular arrhythmias, whereas the malignant arrhythmia-prone category consisted of patients with reduced left ventricular ejection fraction and documented ventricular tachycardia or ventricular fibrillation [22]. 

### Correlation analysis with age and training hours

Correlation analysis of all recorded and calculated standard ECG, HRV, and ventricular heterogeneity parameters (average QRST integral and relative deviation of the QRST integrals) with age and current weekly training hours was also carried out in the group of athletes.

### Subgroup analysis

All recorded and calculated standard ECG, HRV, and ventricular heterogeneity parameters (average QRST integral and relative deviation of the QRST integrals) were also compared in age-matched groups of male and female athletes and controls. Moreover, equal-size age and sex matched groups of athletes doing different sport activities were created based on the sport classification of the ESC [2], and all recorded and calculated standard ECG, HRV, and ventricular heterogeneity parameters were also analysed and compared in these subgroups.

### Statistical analysis

Statistical analyses were performed in Python development environment. Descriptive statistical values are shown as number (percentage) for categorical data, as mean ± SD for normally distributed parameters, as median [interquartile range: 1st quartile–3rd quartile (IQR: Q1–Q3)] for non-normally distributed parameters. Shapiro-Wilk Test, Kurtosis- and Skewness values, and visual assessments (histogram and Q-Q plot) were performed for testing the normality of the parameters. Comprehensive statistical analysis was carried out with unpaired t-test or Wilcoxon rank-sum test (Mann–Whitney U test), depending on the normality of the data. In addition to p-values, between-group effects were quantified using both raw and standardized effect size indices. For non-normally distributed variables, we reported the Hodges–Lehmann estimator (HL) of the location shift (median difference) between groups (athletes minus controls; males minus females), defined as the median of all pairwise differences between observations in the two groups, together with its 95% confidence interval obtained via a nonparametric bootstrap procedure with 5000 resamples. For all continuous outcomes, standardized effect sizes were also expressed as Cohen’s d (standardized mean difference), calculated as the difference between the mean values of the two groups divided by the pooled standard deviation (athletes minus controls; males minus females). Statistical significance was determined if *p* < 0.05. Given the exploratory nature of the multiple secondary and subgroup comparisons, p-values were not formally adjusted for multiple testing; therefore, exact p-values and effect sizes with 95% confidence intervals should be interpreted jointly when considering the strength and practical relevance of the findings. All missing data were proven to be missing-completely random, thereby available-case analysis was carried out for the statistical evaluation. The data underlying this article will be shared on reasonable request to the corresponding author.

## Results

### Participants

We examined 500 elite athletes (age: 19 [16–24] years, male: 55%), who trained an average of 16.7 ± 6.5 h/week. Regarding the sport classification of the ESC, the majority of the examined athletes participated in mixed sports, but athletes from all ESC categories were examined (mixed: 81.9%, power: 9.0%, endurance: 8.8%, skill: 0.3%). Athletes were compared to 100 age and sex matched non-athlete controls who exercised less than 5 h a week (age: 21 [17–23] years, male: 51%).

### Standard ECG parameters

Analysing the measured standard ECG parameters, as expected, resting heart rate was found to be lower in athletes compared to controls. (Table 1) Moreover, significant increases were found in standard ECG lead I P-wave amplitude, PQ duration, QRS width, and T-wave amplitude in athletes as compared to the control group. In contrast, no significant differences in QRS amplitude and QTc duration were found between athletes and controls. (Table 1).

**Table 1:** The measured standard ECG, time domain HRV, and ventricular heterogeneity parameters in athletes and non-athlete controls

	Heart rate (1/min)	*P*-wave amplitude (µV)	PQ duration (ms)	QRS width (ms)	QRS amplitude (µV)	T-wave amplitude (µV)	QTc duration (ms)	AVNN (ms)	SDNN (ms)	RMSSD (ms)	pNN50 (%)	Mean QRST-integral (mV*ms)	QRST-integrals relative deviation
Elite athletes	68 [61–78]	89 [71–108]	152 [140–168]	90 [82–100]	547 [326–777]	257 [198–311]	391 [374–407]	873.4 [770.3-975.9]	64.7 [47.2–87.2]	52.6 [34.8–78.3]	31.1 [12.7–50.9]	34.9 [28.3–43.4]	0.33 [0.22–0.49]
Non-athlete controls	82 [73–91]	80 [62–96]	142 [132–152]	82 [78–90]	465 [269–658]	195 [146–248]	386 [373–400]	740.4 [660.7-828.1]	50.6 [36.5–66.3]	36.4 [26.8–52.6]	14.7 [4.9–31.6]	26.1 [19.7–33.9]	0.33 [0.22–0.48]
*p*	**< 0.001**	**< 0.001**	**< 0.001**	**< 0.001**	0.3	**< 0.001**	0.3	**< 0.001**	**< 0.001**	**< 0.001**	**< 0.001**	**< 0.001**	0.1
HL (CI95%)	**-13** **(-15 to -10)**	**9** **(4 to -15)**	**10** **(6 to14)**	**8** **(4 to 10)**	66 (-8 to 144)	**57** **(39 to 73)**	4 (-1 to 10)	**129.8** **(100.4 to 157.4)**	**14.1** **(9.1 to 18.9)**	**14.6** **(9.8 to 19.1)**	**12.2** **(8.0 to 16.5)**	**9.1** **(7.6 to 11.4)**	0.0 (-0.04 to 0.04)
Cohen’s d	**-0.99**	**0.35**	**0.45**	**0.62**	0.03	**0.60**	0.19	**0.89**	**0.54**	**0.52**	**0.59**	**0.74**	0.02

Effect sizes are presented as athletes minus controls. *Abbreviations*: *AVNN* Average N-N interval, *SDNN* Standard deviation of N-N intervals, *RMSSD* Root mean square of successive N-N differences, *pNN50* Proportion of N-N intervals that differ by at least 50 ms, *HL* Hodges–Lehmann estimator with 95% confidence interval

bold significant difference between the examined groups

### Heart rate variability

**Fig. 1:**
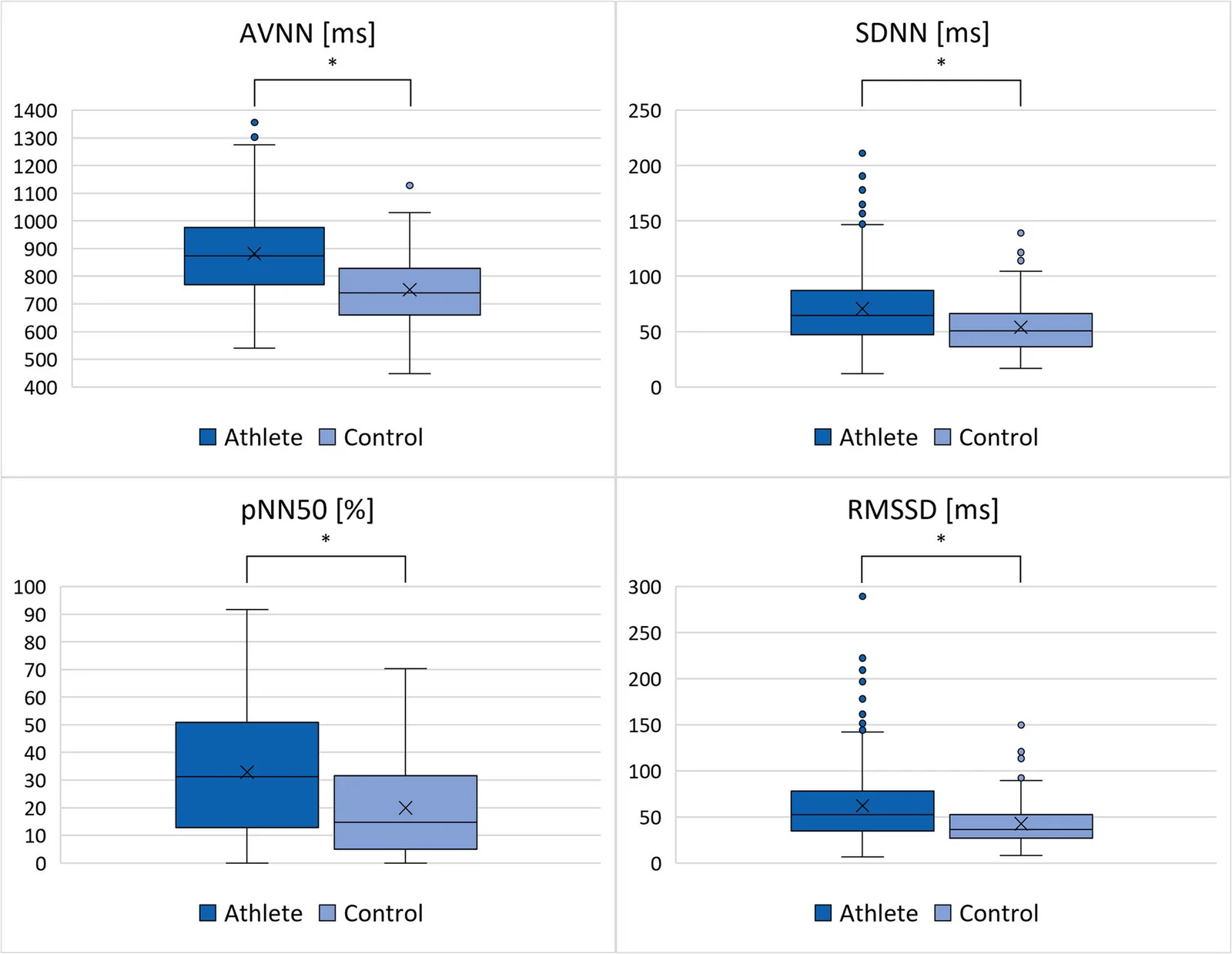
Time domain heart rate variability parameters measured in athletes and non-athlete controls. Abbreviations: AVNN: average N-N interval, SDNN: standard deviation of N-N intervals, RMSSD: root mean square of successive N-N differences, pNN50: proportion of N-N intervals that differ by at least 50 ms, * *p* < 0.001

Examining the time domain parameters of heart rate variability, we found increased values in athletes over controls in AVNN, SDNN, RMSSD, and pNN50. (Fig. 1; Table 1).

### Ventricular heterogeneity

**Fig. 2:**
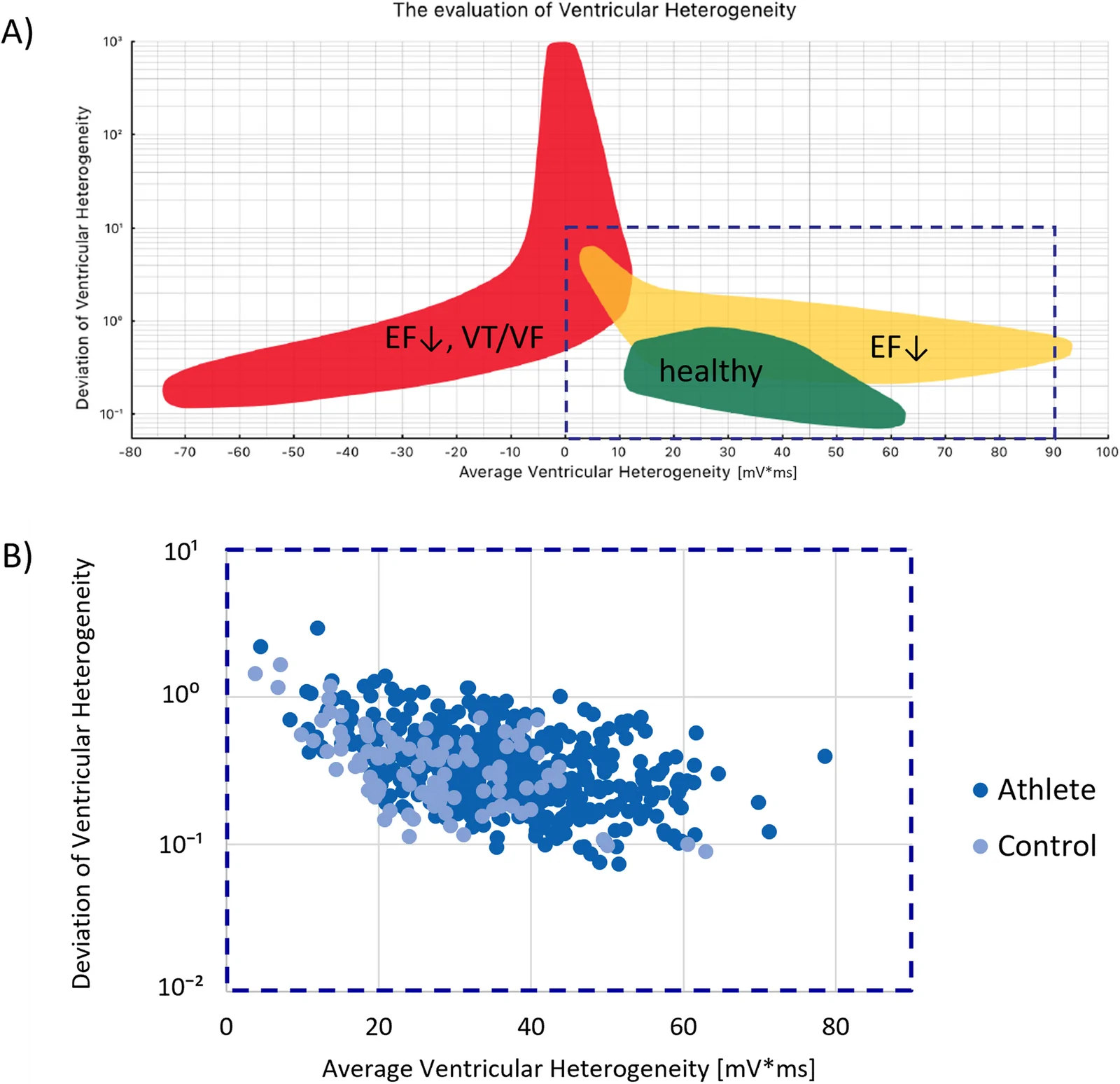
Average and deviation of ventricular heterogeneity. **A** Healthy, intermediate and malignant arrhythmia-prone groups based on previous research by the Wiwe device. Adapted from: [22], green area: healthy; yellow area: intermediate; red area: malignant arrhythmia-prone; blue dashed line: the range our measured values have fallen into (Figure 2B). **B** Ventricular heterogeneity parameters measured in athletes and non-athlete controls. Abbreviations: EF, ejection fraction; VT, ventricular tachycardia; VF, ventricular fibrillation

As for the parameters of ventricular heterogeneity, a significant increase in the mean QRST integral could be observed in athletes compared with controls. However, the relative deviation of QRST integrals did not show any significant difference between the above groups. (Table 1) Regarding the ventricular heterogeneity data, some values of the examined healthy athletes or controls fell within ranges previously associated with arrhythmia populations [22]. (Figure 2A and B)

### Comparing male and female athletes, and male and female controls

We compared data from 208 male athletes (age: 19 [18–21] years) and 154 female athletes (age: 18 [17–22] years). Out of the standard ECG lead I parameters, PQ duration, QRS width, and T-wave amplitude were found to be lower in female athletes as compared to males. In contrast, QTc duration was longer in female athletes in comparison with male athletes. Examining the 1-minute time domain parameters of heart rate variability, no significant differences were found between the two sexes. Regarding the parameters of ventricular heterogeneity, female athletes had a lower mean QRST integral, but a higher relative standard deviation of QRST integrals than male athletes. (Table 2).

**Table 2:** The measured standard ECG, time domain HRV and ventricular heterogeneity parameters in male and female athletes and controls

	Heart rate (1/min)	*P*-wave amplitude (µV)	PQ duration (ms)	QRS width (ms)	QRS amplitude (µV)	T-wave amplitude (µV)	QTc duration (ms)	AVNN (ms)	SDNN (ms)	RMSSD (ms)	pNN50 (%)	Mean QRST- integral (mV*ms)	QRST-integral relative deviation
Male athletes	69 [62–77]	85 [72–101]	154 [142–170]	96 [88–104]	584 [299–813]	260 [209–323]	387 [370–403]	871 [772–961]	62.3 [45.4–83.9]	50.9 [32.1–78.3]	30.5 [10.6–49.1]	36.0 [29.5–44.3]	0.29 [0.21–0.43]
Female athletes	69 [61–80]	89 [72–109]	147 [136–160]	84 [78–92]	480 [289–676]	228 [183–283]	395 [380–414]	864 [752–962]	65.3 [48.2–86.2]	52.0 [37.1–78.6]	29.6 [13.6–51.2]	32.8 [26.3–39.4]	0.38 [0.24–0.50]
*p*	0.43	0.04	**< 0.002**	**< 0.001**	0.12	**< 0.001**	**< 0.001**	0.39	0.47	0.45	0.31	**< 0.001**	**< 0.001**
HL (CI95%)	-1 (-4 to 2)	-2 (-8 to 3)	**6** **(2 to 10)**	**10** **(8 to 12)**	63 (-19 to 136)	**32** **(15 to 48)**	**-10** **(-16 to -5)**	14 (-19 to 46)	-2.4 (-8.2 to 3.9)	-2.5 (-9.1 to 4.0)	-2.4 (-7.1 to 2.2)	**4.1** **(1.7 to 6.4)**	**-0.06** **(-0.10 to -0.03)**
Cohen’s d	-0.12	-0.09	**0.28**	**0.89**	-0.06	**0.42**	**-0.44**	0.11	-0.02	-0.04	-0.10	**0.39**	**-0.18**
Male controls	79 [71–88]	85 [63–96]	144 [134–151]	84 [78–92]	501 [304–714]	211 [150–285]	377 [362–388]	773 [700–846]	52.3 [39.5–70.6]	39.3 [28.4–52.8]	15.7 [5.1–30.9]	28.8 [20.1–38.2]	0.34 [0.22–0.49]
Female controls	84 [76–93]	78 [61–96]	142 [131–152]	82 [77–88]	406 [268–626]	180 [141–221]	394 [383–410]	717 [655–789]	50 [35.3–63.5]	35.2 [25.9–52.2]	12.8 [5.0-31.1]	24.0 [18.6–28.4]	0.38 [0.22–0.48]
*p*	**< 0.04**	0.04	0.68	0.25	0.27	0.07	**< 0.001**	**< 0.03**	0.28	0.46	0.76	**< 0.03**	0.84
HL (CI95%)	**-6** **(-11 to -1)**	4 (-6 to 14)	2 (-6 to 9)	2 (-2 to8)	75 (-58 to 222)	30 (-2 to 61)	**-20** **(-28 to -11)**	**58** **(5 to 105)**	4.8 (-3.5 to 13.0)	2.5 (-4.7 to 10.1)	0.9 (-5.4 to 7.3)	**4.8** **(0.6 to 9.4)**	-0.01 (-0.08 to 0.07)
Cohen’s d	**-0.42**	0.08	0.07	0.33	0.12	0.38	**-0.89**	**0.39**	0.13	0.04	0.00	**0.45**	0.04

Effect sizes are presented as males minus females. *Abbreviations*: *AVNN* Average N-N interval, *SDNN* Standard deviation of N-N intervals, *RMSSD* Root mean square of successive N-N differences, *pNN50* Proportion of N-N intervals that differ by at least 50 ms, *HL* Hodges–Lehmann estimator with 95% confidence interval

bold significant difference between the examined groups

We also compared data from 49 male controls (age: 20 [16–23] years) and 51 female controls (age: 21 [18–24] years). Regarding some examined parameters, we have also found significant differences between male and female controls. Resting heart rate and QTc duration were lower in male controls than in female controls. In addition, we have obtained higher values in AVNN and mean QRST integrals in male controls than in female controls. (Table 2) However, in contrast to the athlete groups, other ECG parameters did not show sex differences in the control group.

### Differences between sport types

Recorded and calculated data of equal-sized age- and sex-matched groups of 50–50 athletes doing power, mixed, and endurance sports were also compared. Regarding the low number of the examined athletes doing skill sports, this group was excluded from this analysis. Significant differences were only found in QRS width between the groups (mixed: 94 [90-101.5] vs. power: 88 [84-95.5] and endurance: 92 [82-101.5] ms, *p* < 0.005). As per the post hoc analysis, the average QRS width was found to be longer in mixed athletes as compared to power athletes.

### Correlation with age and training hours

Average heart rate, P-wave amplitude, QTc duration, and SDNN showed weak negative correlation, while PQ duration, QRS width, and AVNN showed weak positive correlation with age in the athlete group. Average P-wave amplitude, QRS width, QRS amplitude, and QTc duration showed weak positive correlation, while SDNN showed weak negative correlation with weekly training hours in the athlete group. The exact results are shown in Table 3.

**Table 3:** Correlation of the standard ECG, HRV, and ventricular heterogeneity parameters of athletes with age and training hours

Parameters	Correlation with age		Correlation with training hours	
	*r*	*p*	*r*	*p*
Heart rate (1/min)	**-0.204**	**< 0.001**	0.026	0.52
P-wave amplitude (µV)	**-0.15**	**< 0.001**	**0.079**	**< 0.05**
PQ duration (ms)	**0.24**	**< 0.001**	0.037	0.36
QRS width (ms)	**0.144**	**< 0.001**	**0.099**	**< 0.01**
QRS amplitude (µV)	0.024	0.54	**0.101**	**< 0.01**
QTc duration (ms)	**-0.09**	**< 0.05**	**0.087**	**< 0.05**
T-wave amplitude (µV)	-0.021	0.59	0.039	0.32
AVNN (ms)	**0.182**	**< 0.001**	-0.021	0.61
SDNN (ms)	**-0.08**	**< 0.05**	**-0.08**	**< 0.05**
RMSSD (ms)	**-0.115**	**< 0.005**	**-0.105**	**< 0.01**
pNN50 (%)	**-0.107**	**< 0.01**	**-0.098**	**< 0.01**
Mean QRST-integral (mV*ms)	-0.001	0.98	**0.091**	**< 0.02**
QRST-integrals relative deviation	-0.011	0.77	**-0.096**	**< 0.01**

*Abbreviations*: *AVNN* Average N-N interval, *SDNN* Standard deviation of N-N intervals, *RMSSD* Root mean square of successive N-N differences, *pNN50* Proportion of N-N intervals that differ by at least 50 ms

bold significant correlations

## Discussion

Our cross-sectional results suggest that key electrical differences associated with sports adaptation are detectable using an easy-to-record 1-minute lead I standard ECG strip. Regarding the reflected well-known alterations due to the increased muscle mass and chamber volumes, our results are consistent with previous research showing that the athlete’s heart undergoes significant electrical adaptation in response to regular physical exercise [14, 15]. Moreover, our findings indicate that short-term time-dependent changes of the resting ECG in a one-minute recording also mirror the resting sports adaptation changes in the autonomic balance as well as the alterations of the QRST complex referring to ventricular electrical heterogeneity. It is important to emphasize that, as a cross-sectional design, this study can only demonstrate between-group differences at a single time point and does not demonstrate monitoring capability, longitudinal responsiveness, or predictive validity.

Although a standard 12-lead ECG provides considerably more electrophysiological information, including left ventricular hypertrophy voltage criteria, early repolarization, and incomplete right bundle branch block, the purpose of the present study was to investigate whether key markers of electrical sports adaptation can also be detected using a simplified recording approach. Previous work on electrical sports adaptation has relied mainly on standard 12‑lead ECG, extended Holter recordings, or longer HRV protocols, which are well suited for baseline screening or detailed autonomic profiling but less practical for frequent, large‑scale field monitoring in competitive athletes. Our findings indicate that a 1‑minute single‑lead ECG recording can capture not only classical electrical training adaptations but also ultra‑short‑term HRV indices and QRST‑integral‑based markers of ventricular heterogeneity, thereby condensing key information into a simple, portable measurement. In this way, the present approach may help bridge the gap between comprehensive but resource‑intensive methods and the need for scalable, repeatable tools for longitudinal athlete follow‑up.

It is important to emphasize that, although lead I is not among the standard ECG leads showing the most remarkable sports adaptation changes, ECG lead I proved to be suitable for the detection of all the upper detailed electrical sports adaptation changes according to our results. The recording of this lead is easy and quick, and 1-minute recordings can also be carried out by athletes or trainers on the field by using any kind of small portable device. Therefore, this approach may represent a practical and scalable framework for future longitudinal investigations of electrical adaptation in athletes, complementing established methods by providing a simplified, portable measurement of selected electrical adaptation markers.

### Standard ECG changes

Our electrocardiography results confirm that the electrical system undergoes significant changes in trained athletes. Phenomena such as lower resting heart rate, increased P-wave and T-wave amplitude, PQ-interval, and QRS-duration have been previously demonstrated in elite athletes. It is important to emphasize that lead I is not among the standard ECG leads showing the most prominent athletic ECG changes, and a single-lead recording cannot fully characterize the spatial complexity of ventricular depolarisation and repolarisation, including left ventricular hypertrophy voltage criteria, early repolarization, and precordial repolarization patterns. These changes are associated with structural changes like cardiac muscle mass increase and chamber size enlargement, as well as the shift in resting autonomic balance [14, 15, 29]. These findings are further supported by the recent consensus statement of the European Association of Preventive Cardiology (EAPC/ESC), which classifies sinus bradycardia, first-degree atrioventricular block, QRS widening, and increased T-wave amplitude as physiological electrocardiographic manifestations of athletic cardiac adaptation in elite athletes [30]. The novelty in our results regarding standard ECG alterations is that a 1-minute standard ECG lead I recording is also sensitive enough to detect these differences between elite athletes and non-athlete controls.

### Changes in heart rate variability

Long, short, and ultra-short-term methods of measuring heart rate variability are suitable for different purposes, with different advantages and limitations. Long-term HRV measurements (usually 24-hour recordings) accurately reflect the full dynamic spectrum of autonomic nervous system activity, considering also sleep, circadian rhythms, and daily activities. It is thus useful in monitoring general health, chronic stress, and cardiovascular risk assessment, as well as the daylong effects of regular sport activity [18]. In contrast, short- (5-minute) and ultra-short-term (1–3 min) HRV measurements provide quick and easy data collection, especially at rest or in the post-exercise recovery phase, providing information on the actual autonomic nervous system function of the subject [31]. 

All time-domain parameters studied during our ultra-short-term HRV measurements were significantly higher in athletes, indicating increased parasympathetic dominance at rest. This result is in line with previous findings showing that regular physical activity contributes to the favourable regulation of the autonomic nervous system and the shift of autonomic balance toward increased parasympathetic and decreased sympathetic tone in the resting phase [5]. Despite conflicting literature data, and acknowledging the limitations of ultra-short-term recordings, our results suggest that 1-minute time-domain HRV parameters may reflect autonomic adaptations associated with regular athletic training at a between-group level. Whether these parameters provide sufficient reliability for individual-level assessment or serial monitoring requires comparison with standard 5-minute or 24-hour HRV protocols in future studies. In contrast to several complicated techniques applied for the measurement and follow-up of physical fitness [32], our method is easy to use, fast, and reliable. As HRV measurements are also widely used elements of investigating autonomic alterations potentially associated with training load [33], the ease of use of this method can be important in the early detection of this serious condition as well.

### Ventricular heterogeneity

Ventricular heterogeneity reflects the spatial and temporal variability of ventricular depolarisation and repolarisation processes and is considered an indirect marker of myocardial electrical organisation, as the mean QRST integral (reflecting overall ventricular electrical activity) and its beat-to-beat relative deviation (reflecting temporal variability). Increased QTc dispersion connected with the heterogeneity of ventricular repolarisation is known as a marker associated with higher mortality in the general population [34]. The dispersion of QTc was also analysed in athletes, however the results were controversial [35]. In contrast to the QTc dispersion, the QRST integral, a less-known parameter of the ECG, reflects not only the repolarisation, but represents the depolarisation processes of the ventricles as well [36]. Earlier studies have shown that certain changes in ventricular heterogeneity can be connected to severe malignant arrhythmias and thus sudden cardiac death [20]. The degree of ventricular heterogeneity can be assessed by calculating the QRST integral [37]. It should be acknowledged that ventricular heterogeneity is a spatially distributed electrophysiological phenomenon. Therefore, the present lead I–based approach provides only a simplified representation of ventricular electrical heterogeneity and cannot fully replace comprehensive multi-lead assessment.

A previous study has shown that a negative shift in the QRST integral could also be associated with malignant ventricular arrhythmias [38], but such studies have not been performed in athletes yet. Besides the importance of early recognition of the susceptibility to malignant ventricular arrhythmias, another intriguing question is whether ventricular heterogeneity and QRST integral show sports adaptation changes.

Our results suggest that the answer is yes, as the mean QRST integral proved to be higher in athletes. This result could be related to the increased heart muscle mass as well as the electrical remodelling induced by regular physical exercise. However, the relative deviation of the QRST integrals did not show significant variance as compared to the healthy non-athlete population. As the decrease of mean QRST integral, as well as the increase of the relative deviation of the QRST integrals were formerly seen in the patient group having the diagnosis of previous malignant ventricular arrhythmias [22], our results refer to the favourable fact that ventricular heterogeneity changes due to sports adaptation do not increase arrhythmic risk. Our results are in contrast with some previous data proposing the increased number of ventricular arrhythmias as a part of sports adaptation processes in healthy athletes [39]. 

In this context, it is important to highlight that regular evaluation of ventricular heterogeneity can contribute to the follow-up of the athletes and, more importantly, to the early detection of pathological changes and increased malignant arrhythmia risk. Some participants exhibited QRST-integral values that fell within ranges previously associated with populations affected by malignant ventricular arrhythmias, despite the otherwise negative screening results. These results require further investigations, and these individuals need thorough cardiology follow-up for the potentially increased risk of malignant ventricular arrhythmias. All of these individuals underwent additional Holter ECG evaluation as part of their cardiology assessment. No clinically relevant ventricular arrhythmias or other findings supporting increased arrhythmic risk were detected during these examinations. We recommended that these individuals undergo regular cardiological checkups even if they are asymptomatic. However, no arrhythmic outcomes, longitudinal follow-up, or clinical validation data are available in the present cohort; therefore, no exact conclusions regarding individual arrhythmic risk can be drawn.

### Differences between male and female athletes and controls

First of all, our study revealed that some sex-related differences are already present in non-athlete individuals as well. For instance, resting heart rate and AVNN were higher, and QTc duration was longer in female controls compared to male controls. In addition, male controls had higher mean QRST integral values than females. Therefore, not all observed differences between male and female athletes can be attributed exclusively to the effects of training; some are likely due to innate physiological differences between the sexes.

However, many parameters showed gender differences exclusively in athletes but not in controls, suggesting that these may reflect sex-related patterns of cardiac adaptation to regular training. Specifically, PQ duration, QRS width, and T-wave amplitude were significantly lower in female athletes compared to male athletes, while no such differences were observed among controls. Additionally, female athletes had a higher relative standard deviation of QRST integrals, which was not present in the control group. These findings may point to exercise-induced gender differences in electrical cardiac remodelling. However, in contrast to the controls, higher resting heart rates of females could not be observed in athletes.

In terms of sex differences, female athletes show less pronounced electrical sports adaptation signals as compared to males, according to our study regarding the standard ECG and the mean QRST integral values. This is in accordance with the results of many other human studies suggesting that structural and functional changes of the heart, as well as ECG alterations are more pronounced in male athletes than in females [29, 40]. The gender differences detailed above are probably based on neuroendocrine, metabolic, and cardiac morphological differences. Research has shown that hormonal differences between male and female individuals play a significant role in cardiovascular responses to exercise. As the most known phenomenon, oestrogen exerts a protective effect on the cardiovascular system, affecting blood pressure regulation, heart rate, and vascular function [41]. Besides affecting cardiovascular processes, hormonal differences between sexes also have an impact on metabolism and energy usage. Female athletes tend to rely more on lipid metabolism during endurance training, while male athletes are more likely to obtain energy through glycolytic processes [42]. In terms of cardiac morphology, research has revealed that male athletes have a generally larger left ventricular mass, larger ventricular cavities, and greater wall thickness when adjusted for body surface area than female athletes [40, 43]. These factors may also contribute to the sex differences in electrical sports adaptation found by our research, but further studies are required to find out the exact mechanisms behind down to the level of cardiac ion channels.

In contrast to the above, our ultra-short-term time domain HRV parameters did not show sex differences. Sex differences in autonomic sports adaptation are a special area of sports cardiology and require further research, but there have been some previous studies that have investigated autonomic nervous system differences between male and female athletes [44, 45]. Although, unlike our study, they did not use ultra-short-term HRV recordings, but rather long-term HRV measurements. Therefore, these studies mainly analysed frequency-domain indicators (e.g., high-frequency and low-frequency components) of HRV [26]. These studies have consistently found that female athletes have a significantly higher high-frequency HRV component, indicating an increased activity of the parasympathetic nervous system, including the vagus nerve. On the other hand, in male athletes, the low-frequency component dominated, suggesting a stronger influence of the sympathetic nervous system. This difference in HRV could also be observed at rest and was maintained during exercise, and may even increase after training. It is not completely understood which factors are responsible for these variations, but the hormonal differences that have been mentioned earlier definitely play an important role in this process. The fact that these HRV differences are significantly reduced in females over the age of 50, having lower levels of oestrogen, seems to confirm this theory [44]. In addition, males have a larger muscular mass and also have more neurons in the sympathetic ganglions than females, which could also contribute to the increased sympathetic nervous activity of males [44]. However, our ultra-short-term HRV measurements did not mirror these differences. Theoretically, the increase of the sympathetic tone due to the psychological stress experienced in the clinic could also obscure these differences.

The higher relative standard deviation of QRST integrals in female athletes compared to males is another fascinating finding of our study. Although the mechanisms behind this need to be explored in detail in the future, it is conceivable that, since the QTc-interval is also longer in females, this may also be explained by differences in the repolarisation processes of the myocytes [46]. 

### Influence of sport types

The different physical workloads required by different sports trigger diverse adaptation mechanisms in the heart, both structurally and functionally. As it has been described previously, there is a marked eccentric left ventricular hypertrophy in dynamic sports, whereas there is less pronounced cardiac adaptation in power sports. In mixed sport types, which include both dynamic and static components, a combined adaptation appears [8, 47]. These adaptations are not only reflected in morphological changes of the heart, but they are also followed by changes in the electrocardiogram. Our measurements have verified that mixed athletes have a significantly wider QRS complex compared to power athletes, demonstrating the difference in adaptation processes, possibly connected to the higher muscle mass of the mixed athlete group. However, no significant difference was found between endurance athletes and mixed athletes, as well as endurance athletes and power athletes regarding the QRS width. Moreover, no difference was found between the groups regarding other examined parameters. These results show that our method, although fast and easy to use, is not suitable for recognizing subtle differences in sports adaptation to different sports. Enlarging the examined athlete groups, increasing the recording time, or increasing the number of examined ECG leads could help to analyse less pronounced differences in electrical sports adaptation processes of the heart regarding different sports activities.

Because the distribution of sport categories was uneven and some subgroups were relatively small, these findings should be considered exploratory and interpreted with caution until confirmed in larger and more balanced cohorts.

### Age and training hours

Examining the effects of age and weekly training hours on electrical sports adaptation processes, weak correlations were found regarding certain ECG, HRV, and ventricular heterogeneity signs of sports adaptation. It has been shown previously that the type of sport load, intensity and number of years in sport participation play a more significant role in adaptation processes than actual training hours or age [48] Therefore, we would like to extend this research in the future with a multi-parameter study to get a clearer picture of which parameters are the major determinants of the resting electrical parameters of the athletic heart. Also, it would be worthwhile to examine the cost-effectiveness and economic aspects of long-term ECG monitoring of athletes. With the current setup, a single device could serve multiple athletes, thereby reducing the per capita cost of a single device.

Although artificial intelligence-based ECG analysis was not applied in the present study, the availability of standardized digital single-lead ECG recordings may provide a suitable basis for future machine-learning and deep-learning approaches aimed at individualized athlete monitoring, automated arrhythmia detection, and differentiation between physiological and pathological electrical remodelling. Recent developments in cardiac electrophysiology have highlighted the growing role of artificial intelligence in ECG interpretation and risk stratification [49]. 

### Limitations

The majority of the examined athletes were participating in mixed sports. Moreover, the median age of our athletes was 19 years, so our results are relevant among young athletes. Therefore, further extended investigations would be necessary to see how the measured parameters evolve in older athlete populations and in cases of sport activities not yet studied in large numbers.

Another limiting factor of our research is also an important advantage of it. The fact that we used a mobile device recording lead I ECG for our measurements limited our ability to investigate all aspects of electrical adaptation; however, this easily portable and reproducible method of recording and analysis can be easily integrated into the everyday lives of athletes and regular field measurements. Furthermore, ventricular heterogeneity is inherently spatial in nature, and therefore, a single-lead recording cannot fully characterise the three-dimensional electrical properties of ventricular depolarisation and repolarisation. Moreover, any portable device that can detect and store ECG data could be suitable to reproduce our results. Certainly, the detection of all 12 leads in a further study, as well as analysing follow-up measurements and longitudinal examinations of the same athletes, could help us to even better understand the electrical processes ongoing in the athlete’s heart. Our follow-up measurements are already ongoing.

Ultra-short-term HRV measurements represent a practical and easily applicable fast method; however, they are more sensitive to physiological and environmental influences than conventional 5-minute or 24-hour recordings. Respiratory pattern, hydration status, sleep quality, mental stress, and other autonomic influences were not directly controlled or measured in the present study. Therefore, HRV findings should be interpreted within the limitations of ultra-short-term recordings.

Several ECG parameters are influenced by anthropometric characteristics, including body size, body surface area, body composition, and cardiac dimensions. These variables were not incorporated into multivariable analyses in the present study; therefore, the independent contribution of sex-related and training-related factors cannot be fully determined. The present study does not include a direct within-cohort comparison of the examined parameters with standard 12-lead ECG, 5-minute or 24-hour HRV recordings, echocardiographic measurements, cardiac MRI, or cardiopulmonary exercise testing results. Such validation would be necessary to establish independent diagnostic value and is a priority for future research. Moreover, given substantial differences in heart rate between athletes and controls, Bazett correction may introduce bias.

The present study was not designed to evaluate long-term clinical outcomes, arrhythmia incidence, sudden cardiac arrest, or sudden cardiac death. Therefore, no conclusions regarding future clinical events can be drawn at the current state.

Although several correlations reached statistical significance, their magnitude was generally weak and should therefore be interpreted cautiously. The statistical significance likely reflects the large sample size rather than strong physiological relationships. Moreover, multiple comparisons were conducted during the statistical analysis, which could increase the statistical uncertainty.

## Conclusion

The present cross-sectional study demonstrates that 1-minute single-lead I ECG recordings can detect significant electrical differences between elite athletes and non-athlete controls and could be suitable for detecting the electrical processes of the athlete’s heart. Standard ECG parameters, time-domain HRV indices, and QRST-integral–derived markers of ventricular heterogeneity differed significantly between the examined groups. These findings are consistent with known structural, functional, metabolic, and autonomic sports adaptation processes. Based on our results, these electrical changes show marked differences regarding male and female athletes, while the influence of sports activity, age, and training hours requires further evaluation. These measurements can be easily incorporated into routine athlete screening programmes as a complementary, rapid assessment tool. Future longitudinal studies are required to determine whether serial measurements of these parameters can provide clinically meaningful information regarding training adaptation, fitness status, arrhythmia development, or overtraining. Moreover, future studies integrating structural and electrical parameters may also provide additional insights into the complex interrelationships in sports adaptation processes.

## Data Availability

The datasets used and analysed during the current study are available from the corresponding author on reasonable request.

## Abbreviations

HRV: Heart rate variability
ESC: European Society of Cardiology
AVNN: Average N-N interval
SDNN: Standard deviation of N-N intervals
RMSSD: Root mean square of successive N-N differences
pNN50: Proportion of N-N intervals that differ by at least 50 milliseconds from each other compared to the total NN intervals
HL: Hodges-Lehmann estimator

## References

[1] Hughes DC, Ellefsen S, Baar K. Adaptations to Endurance and Strength Training. Cold Spring Harb Perspect Med. 2018;8(6). 10.1101/cshperspect.a029769. PMC5983157 28490537

[2] Pelliccia A, Sharma S, Gati S, Bäck M, Börjesson M, Caselli S, et al. 2020 ESC Guidelines on sports cardiology and exercise in patients with cardiovascular disease: The Task Force on sports cardiology and exercise in patients with cardiovascular disease of the European Society of Cardiology (ESC). Eur Heart J. 2020;42(1):17–96. 10.1093/eurheartj/ehaa605 32860412

[3] Zamodics M, Babity M, Schay G, Leel-Ossy T, Bucsko-Varga A, Kulcsar P, et al. Correlations Between Body Composition and Aerobic Fitness in Elite Female Youth Water Polo Players. Sports. 2025;13(2):51. 10.3390/sports13020051 PMC11861686 39997982

[4] Schuch FB, Stubbs B. The Role of Exercise in Preventing and Treating Depression. Curr Sports Med Rep. 2019;18(8):299–304. 10.1249/JSR.0000000000000620 31389872

[5] Seravalle G, Grassi G. Effect of Regular Exercise on Autonomic Nervous System Activity. In: Palatini P, Agabiti-Rosei E, Mancia G, editors. Exercise, Sports and Hypertension. Cham: Springer International Publishing; 2022. pp. 31–42.

[6] Carter JB, Banister EW, Blaber AP. Effect of endurance exercise on autonomic control of heart rate. Sports Med. 2003;33(1):33–46. 10.2165/00007256-200333010-00003 12477376

[7] Aune D, Norat T, Leitzmann M, Tonstad S, Vatten LJ. Physical activity and the risk of type 2 diabetes: a systematic review and dose-response meta-analysis. Eur J Epidemiol. 2015;30(7):529–42. 10.1007/s10654-015-0056-z 26092138

[8] Niebauer J, Börjesson M, Carre F, Caselli S, Palatini P, Quattrini F, et al. Recommendations for participation in competitive sports of athletes with arterial hypertension: a position statement from the sports cardiology section of the European Association of Preventive Cardiology (EAPC). Eur Heart J. 2018;39(40):3664–71. 10.1093/eurheartj/ehy511 30165596

[9] Lakatos BK, Kiss O, Tokodi M, Toser Z, Sydo N, Merkely G, et al. Exercise-induced shift in right ventricular contraction pattern: novel marker of athlete’s heart? Am J Physiol Heart Circ Physiol. 2018;315(6):H1640–8. 10.1152/ajpheart.00304.2018 30216120

[10] D’Ascenzi F, Anselmi F, Focardi M, Mondillo S. Atrial Enlargement in the Athlete’s Heart: Assessment of Atrial Function May Help Distinguish Adaptive from Pathologic Remodeling. J Am Soc Echocardiogr. 2018;31(2):148–57. 10.1016/j.echo.2017.11.009 29246514

[11] Balla D, Szabo L, Graziano F, Mesko C, Dohy Z, Juhasz V, et al. The role of sex, training load, and sports type in athletic cardiac remodelling: Insights from T1 and T2 mapping via cardiac magnetic resonance. Int J Cardiol. 2025;426:133080. 10.1016/j.ijcard.2025.133080 39983876

[12] Rafla S, Elzawawy T, Mahmoud G, Sadaka M, Salam A. Study of electrocardiogram in competitive athletes. Biolife. 2017;5(4):474–8. 10.5281/zenodo.7389621.

[13] Zamodics M, Babity M, Schay G, Bucsko-Varga A, Kovacs E, Horvath M, et al. Investigation of Body Composition and Cardiac Sports Adaptation in Elite Water Polo Players. Sports. 2025;13(6):180. 10.3390/sports13060180 PMC12196840 40559692

[14] Sharma S, Drezner JA, Baggish A, Papadakis M, Wilson MG, Prutkin JM, et al. International recommendations for electrocardiographic interpretation in athletes. Eur Heart J. 2017;39(16):1466–80. 10.1093/eurheartj/ehw631 28329355

[15] Kiss O, Sydó N, Vargha P, Édes E, Merkely G, Sydó T, et al. Prevalence of physiological and pathological electrocardiographic findings in Hungarian athletes. Acta Physiol Hung. 2015;102(2):228–37. 10.1556/036.102.2015.2.13 26100312

[16] Rafla S, El Zawawy T, Sadaka M, Abd Elnaser G, Nassar A. Study of the value of routine electrocardiogram in competitive athletes. EP Europace. 2017;19(suppl3):iii332–iii.

[17] Rafla S, Younes G, Nassar A, Kamal A. The Significance of early repolarization and incomplete right bundle block in athletes. Int J Sports Exerc Med. 2020;6(3). 10.23937/2469-5718/1510168.

[18] Kiss O, Sydo N, Vargha P, Vago H, Czimbalmos C, Edes E, et al. Detailed heart rate variability analysis in athletes. Clin Auton Res. 2016;26(4):245–52. 10.1007/s10286-016-0360-z 27271053

[19] Dong JG. The role of heart rate variability in sports physiology. Exp Ther Med. 2016;11(5):1531–6. 10.3892/etm.2016.3104 PMC4840584 27168768

[20] Tereshchenko LG. Global Electrical Heterogeneity: Mechanisms and Clinical Significance. Comput Cardiol (2010). 2018;45. 10.22489/cinc.2018.165. PMC7158901 32296725

[21] Kozmann G, Tuboly G, Szathmáry V, Švehlíková J, Tyšler M. Computer modelling of beat-to-beat repolarization heterogeneity in human cardiac ventricles. Biomed Signal Process Control. 2014;14:285–90.

[22] Kozmann G, Tuboly G, Tarjányi Z, Szathmáry V, Švehlíková J, Tyšler M. Model interpretation of body surface potential QRST integral map variability in arrhythmia patients. Biomed Signal Process Control. 2014;12:3–9.

[23] Tuboly G, Kozmann G, Kiss O, Merkely B. Pitvarfibrilláció detektálása szívritmus és EKG hullámforma alapján. Interdiszciplináris Magyar Egészségügy. 2017;XVI(6):46–9.

[24] Sanatmetal Ltd. References for our algorithm’s accuracy for detecting atrial fibrillation. Available from: https://www.mywiwe.com/files/PDF/clinical_AF_hu.pdf. [Cited 2026. 06.21.].

[25] NEOEMKI Nemzeti Orvostechnikai Eszköz Megfelelőségértékelő. és Tanúsító Kft. EK-Tanúsítvány 2009. Available from: https://www.mywiwe.com/files/adatvedelem_html/NewFolder/EK%20Tanúsítvány%20-%20WIWE.pdf. [Cited 2026. 06. 21.].

[26] Heart rate variability. : standards of measurement, physiological interpretation and clinical use. Task Force of the European Society of Cardiology and the North American Society of Pacing and Electrophysiology. Circulation. 1996;93(5):1043–65. 8598068

[27] Geselowitz DB. The ventricular gradient revisited: relation to the area under the action potential. IEEE Trans Biomed Eng. 1983;30(1):76–7. 10.1109/tbme.1983.325172 6826192

[28] Tuboly G, Kiss O, Babity M, Zamodics M, Merkely B, Kozmann G, et al. Malignant arrhythmia risk assessment based on lead-I mobile ECG measurements using machine learning. J Electrocardiol. 2026;95:154194. 10.1016/j.jelectrocard.2026.154194 41570464

[29] D’Ascenzi F, Cavigli L, Marchese A, Taddeucci S, Cappelli E, Roselli A, et al. Electrical and structural remodelling in female athlete’s heart: A comparative study in women vs men athletes and controls. Int J Cardiol. 2024;400:131808. 10.1016/j.ijcard.2024.131808 38262482

[30] Finocchiaro G, Zorzi A, Abela M, Baggish A, Castelletti S, Cavarretta E, et al. Abnormal electrocardiogram findings in athletes. Eur Heart J. 2026;47(2):152–69. 10.1093/eurheartj/ehaf646 PMC12777699 40996312

[31] Jin K, Guo Z, Qiao Z, Liu M, Yang Y, Xu C. Agreement between Ultra-Short-Term and Standard Heart Rate Variability Analysis in Resting and Post-Exercise Conditions. Life. 2024;14(7):837. 10.3390/life14070837 PMC11278104 39063591

[32] Hollmann W, Knigge H, Knicker A, Strüder HK. Methods for Measurement of Physical Fitness and Training Recommendations in Studies on Humans. In: Boecker H, Hillman CH, Scheef L, Strüder HK, editors. Functional Neuroimaging in Exercise and Sport Sciences. New York, NY: Springer New York; 2012. pp. 79–107.

[33] Hynynen E, Uusitalo A, Konttinen N. Heart rate variability during night sleep and after awakening in overtrained athletes. Med Sci Sports Exerc. 2006;38(2):313–7. 10.1249/01.mss.0000184631.27641.b5 16531900

[34] de Bruyne MC, Hoes AW, Kors JA, Hofman A, van Bemmel JH, Grobbee DE. QTc dispersion predicts cardiac mortality in the elderly: the Rotterdam Study. Circulation. 1998;97(5):467–72. 10.1161/01.cir.97.5.467 9490242

[35] Stoičkov V, Radovanović D, Deljanin-Ilić M, Perišić Z, Pavlović M, Tasić I, et al. Sport-related differences in QT dispersion and echocardiographic parameters in male athletes. Sci Rep. 2023;13(1):6770. 10.1038/s41598-023-33957-8 PMC10130053 37185606

[36] Sur S, Han L, Tereshchenko LG. Comparison of Sum Absolute QRST Integral, and Temporal Variability in Depolarization and Repolarization, Measured by Dynamic Vectorcardiography Approach, in Healthy Men and Women. PLoS ONE. 2013;8(2):e57175. 10.1371/journal.pone.0057175 PMC3579786 23451181

[37] Tereshchenko LG, Cheng A, Fetics BJ, Marine JE, Spragg DD, Sinha S, et al. Ventricular arrhythmia is predicted by sum absolute QRST integralbut not by QRS width. J Electrocardiol. 2010;43(6):548–52. 10.1016/j.jelectrocard.2010.07.013 PMC2970680 20832820

[38] György PDK, Gergely T, Zoltán KG, Orsolya DK, Béla PDM. Kardiovaszkuláris rizikótényezők okostelefonos vizsgálata. 2016.

[39] Heidbuchel H, Prior DL, La Gerche A. Ventricular arrhythmias associated with long-term endurance sports: what is the evidence? Br J Sports Med. 2012;46(Suppl 1):i44–50. 10.1136/bjsports-2012-091162 23097479

[40] Csecs I, Czimbalmos C, Toth A, Dohy Z, Suhai IF, Szabo L, et al. The impact of sex, age and training on biventricular cardiac adaptation in healthy adult and adolescent athletes: Cardiac magnetic resonance imaging study. Eur J Prev Cardiol. 2020;27(5):540–9. 10.1177/2047487319866019 31370686

[41] Marongiu E, Crisafulli A. Gender differences in cardiovascular functions during exercise: a brief review. Sport Sci Health. 2015;11(3):235–41.

[42] Wohlgemuth KJ, Arieta LR, Brewer GJ, Hoselton AL, Gould LM, Smith-Ryan AE. Sex differences and considerations for female specific nutritional strategies: a narrative review. J Int Soc Sports Nutr. 2021;18(1):27. 10.1186/s12970-021-00422-8 PMC8015182 33794937

[43] Giraldeau G, Kobayashi Y, Finocchiaro G, Wheeler M, Perez M, Kuznetsova T, et al. Gender Differences in Ventricular Remodeling and Function in College Athletes, Insights from Lean Body Mass Scaling and Deformation Imaging. Am J Cardiol. 2015;116(10):1610–6. 10.1016/j.amjcard.2015.08.026 26456207

[44] Dutra SGV, Pereira APM, Tezini GCSV, Mazon JH, Martins-Pinge MC, Souza HCD. Cardiac Autonomic Modulation Is Determined by Gender and Is Independent of Aerobic Physical Capacity in Healthy Subjects. PLoS ONE. 2013;8(10):e77092. 10.1371/journal.pone.0077092 PMC3789672 24098577

[45] Kappus RM, Ranadive SM, Yan H, Lane-Cordova AD, Cook MD, Sun P, et al. Sex differences in autonomic function following maximal exercise. Biology Sex Differences. 2015;6(1):28. 10.1186/s13293-015-0046-6 PMC4666049 26629325

[46] Tereshchenko L. QT variability and QRST integral. 2020. pp. 117 – 23.

[47] Morrison BN, George K, Kreiter E, Dixon D, Rebello L, Massarotto RJ, et al. Effects of endurance exercise training on left ventricular structure in healthy adults: a systematic review and meta-analysis. Eur J Prev Cardiol. 2023;30(9):772–93. 10.1093/eurjpc/zwad023 36718569

[48] Luijkx T, Cramer MJ, Prakken NHJ, Buckens CF, Mosterd A, Rienks R, et al. Sport category is an important determinant of cardiac adaptation: an MRI study. Br J Sports Med. 2012;46(16):1119–24. 10.1136/bjsports-2011-090520 22219218

[49] Cipollone P, Pierucci N, Matteucci A, Palombi M, Laviola D, Bruti R et al. Artificial Intelligence in Cardiac Electrophysiology: A Comprehensive Review. J Pers Med. 2025;15(11). 10.3390/jpm15110532. PMC12653158 41295237

